# Conserved Conformational Hierarchy across Functionally Divergent Glycosyltransferases of the GT-B Structural Superfamily as Determined from Microsecond Molecular Dynamics

**DOI:** 10.3390/ijms22094619

**Published:** 2021-04-28

**Authors:** Carlos A. Ramirez-Mondragon, Megin E. Nguyen, Jozafina Milicaj, Bakar A. Hassan, Frank J. Tucci, Ramaiah Muthyala, Jiali Gao, Erika A. Taylor, Yuk Y. Sham

**Affiliations:** 1 Bioinformatics and Computational Biology Program, University of Minnesota, Minneapolis, MN 55455, USA; rami0255@umn.edu (C.A.R.-M.); nguy1892@umn.edu (M.E.N.); gao@jialigao.org (J.G.); 2 Department of Chemistry, Wesleyan University, Middletown, CT 06459, USA; jmilicaj@wesleyan.edu (J.M.); bhassan@wesleyan.edu (B.A.H.); Ftucci@wesleyan.edu (F.J.T.); 3 Department of Experimental and Clinical Pharmacology, College Pharmacy, University of Minnesota, Minneapolis, MN 55455, USA; muthy003@umn.edu; 4 Department of Chemistry, University of Minnesota, Minneapolis, Minneapolis, MN 55455, USA; 5 Institute of Systems and Physical Biology, Shenzhen Bay Laboratory, Shenzhen 518055, China; 6 Department of Integrative Biology and Physiology, Medical School, University of Minnesota, Minneapolis, MN 55455, USA

**Keywords:** GT, glycosyltransferase, HepI, heptosyltransferase I, heptose, L-*glycero*-d-*manno*-heptose, ADP-heptose or ADPH, ADP-L-*glycero*-*D*-*manno*-heptose, LPS, lipopolysaccharide, ODLA, O-deacylated *E. coli* Kdo_2_-lipid A, CD, circular dichroism, MD, molecular dynamics, PDB, Protein Data Bank, PCA, principal component analysis, DCC, dynamic cross-correlation

## Abstract

It has long been understood that some proteins undergo conformational transitions en route to the Michaelis Complex to allow chemistry. Examination of crystal structures of glycosyltransferase enzymes in the GT-B structural class reveals that the presence of ligand in the active site triggers an open-to-closed conformation transition, necessary for their catalytic functions. Herein, we describe microsecond molecular dynamics simulations of two distantly related glycosyltransferases that are part of the GT-B structural superfamily, HepI and GtfA. Simulations were performed using the open and closed conformations of these unbound proteins, respectively, and we sought to identify the major dynamical modes and communication networks that interconnect the open and closed structures. We provide the first reported evidence within the scope of our simulation parameters that the interconversion between open and closed conformations is a hierarchical multistep process which can be a conserved feature of enzymes of the same structural superfamily. Each of these motions involves of a collection of smaller molecular reorientations distributed across both domains, highlighting the complexities of protein dynamic involved in the interconversion process. Additionally, dynamic cross-correlation analysis was employed to explore the potential effect of distal residues on the catalytic efficiency of HepI. Multiple distal nonionizable residues of the C-terminal domain exhibit motions anticorrelated to positively charged residues in the active site in the *N*-terminal domain involved in substrate binding. Mutations of these residues resulted in a reduction in negatively correlated motions and an altered enzymatic efficiency that is dominated by lower K_m_ values with *k*_cat_ effectively unchanged. The findings suggest that residues with opposing conformational motions involved in the opening and closing of the bidomain HepI protein can allosterically alter the population and conformation of the “closed” state, essential to the formation of the Michaelis complex. The stabilization effects of these mutations likely equally influence the energetics of both the ground state and the transition state of the catalytic reaction, leading to the unaltered *k*_cat_. Our study provides new insights into the role of conformational dynamics in glycosyltransferase’s function and new modality to modulate enzymatic efficiency.

## 1. Introduction

Glycosylation is a highly regulated, ubiquitous biochemical process catalyzed by glycosyltransferase (GT, E.C. 2.4.x.y) enzymes. The implications of glycosylation in cellular processes are broad, as it modulates the structure, stability, and hence function of the target molecule. Although GTs constitute approximately 1 to 2% of the genomes that have been sequenced from species across living kingdoms [1], the details of the molecular mechanism of many GTs remain poorly understood. Significant research endeavors have identified and characterized key residues and regions linked to the enzymatic cycle in a diverse range of GTs [2,3,4,5,6,7,8,9]. These investigations provided a wealth of data, making it possible to develop an atomistic description of the molecular mechanism of GTs. An understanding of the functional dynamics of this important class of enzymes is paramount for inhibitor discovery, chimeric protein design, enzymatic control and regulation, among other studies that offer potentially beneficial clinical and industrial applications.

GTs mediate the transfer of a single sugar (monosaccharide) from an activated sugar complex (sugar donor) onto a specific acceptor substrate [10]. The identities of the substrates (both the sugar donor and acceptor) are GT-specific, with the pool of available sugar donors encompassing a myriad of monosaccharides and activation moiety conjugates (with the latter including nucleotide-monophosphate, nucleotide-diphosphate, lipid-phosphate, or unsubstituted-phosphate compounds) [10]. The sugar acceptor substrates range from single biomolecules to macromolecular complexes of varying compositions (e.g., monosaccharides, oligosaccharides, organic molecules, lipids, polypeptides, DNA) [10]. The resulting sugar conjugate, in most reported cases, serves as the preferred substrate for a subsequent GT in a series of glycosylation reactions that lead to the final multiglycosylated product [3,4,5,11].

**Figure 1:**
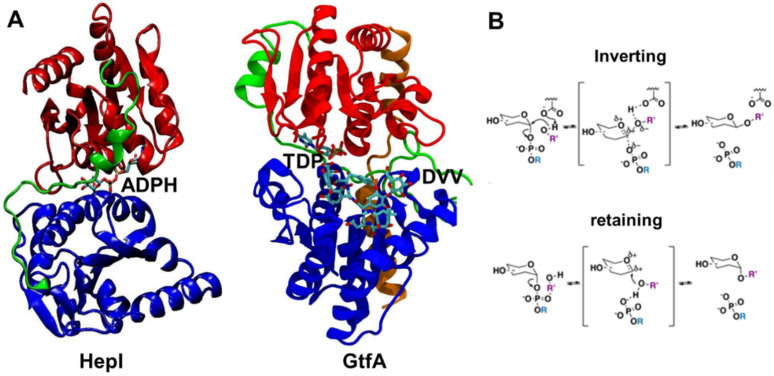
Crystal structures of ligand-bound glycosyltransferases: (**A**) HepI (PDB: 2H1H) and GtfA (PDB: 1PN3) colored for N domain (blue), *C* domain (red), linker (green), and spine (orange, only in GtfA) regions. HepI and GtfA cofactors, ADPH and TDP, respectively, and GtfA substrate DVV, are shown as ball and stick representation, which are located between *N* and *C* domains. Ligands are included to highlight the location of the binding catalytic site. (**B**) Inverting and retaining mechanisms by glycosyltransferases. Our simulations were performed without any substrates for the unbound HepI and GtfA.

The largest subset of identified GTs in the Carbohydrate-active enzyme (CAZy) database corresponds to the bidomain Leloir-GTs (monosaccharide-nucleotide dependent) [12]. The GTs in the CAZy database have been classified into 111 different enzyme families based upon their primary sequences, with individual domains of Leloir-GTs named according to their relative primary sequence arrangement (*N* and *C* domains), while their combined spatial configurations determine the structural superfamily (GT-A, GT-B, among others) [13]. Regardless of the structural superfamily classification, Leloir-GT structures depict the nucleotide binding Rossman fold (βαβ) motif as a conserved feature that comprises the core of all resolved domains. In GT-A proteins, two Rossman domains are aligned to create a seven-stranded β-sheet at the core of the proteins which undergoes modest structural rearrangements over the course of the transfer reaction, mostly the result of loop region movements [14,15]. GT-B glycosyltransferases such as Heptosyltransferase I (HepI) and TDP-*epi*-vancosaminyltransferase (GtfA) consist of topologically identical Rossman-like domains joined by an extended linker (loop-α-loop) region, also known as the spine region, that are positioned in a stacked configuration (Figure 1). This arrangement generates a central inter-domain cavity that serves as the binding site for both ligands and contains the enzyme’s catalytic core. The binding position of each ligand in the GT-B cavity is domain-specific, as the *N* and *C* domains demonstrate selective binding affinity towards the acceptor substrate and sugar donor substrate, respectively, and the two domains undergo conformational transitions to bring the two domains proximal prior to catalysis [3,4,5]. The location of the ligands in the binding pocket further divides the cavity into catalytic (proximal to active site residues) and non-catalytic regions. GTs are further classified mechanistically as either configuration-retaining or configuration-inverting (Figure 1) [10]. The classification is given by comparing the stereochemistry of the newly formed glycosidic linkage of the product to that of the starting sugar donor substrate.

As observed experimentally, the transition from a catalytically inactive (open) to active (closed ternary) state in GT-Bs is induced by substrate binding and is proposed to proceed via domain motions including hinge-bending centered on residues of the linker and/or the extended C-terminal spine region (if present), as well as twists that align the two substrates [4,5,11,16]. These motions are accompanied by different degrees of peripheral α-helix repositioning and loop restructuring with no significant alteration to the underlying β-sheet of the Rossman fold of each domain [4,5]. The end result of this dynamic process, described here as domain flexibility, positions the domains closer to one another, emphasizing pre-existing or creating new intra- and inter-domain contacts while bridging the distance between the enzymatic and ligand reactive centers [4,5]. Crystallographic examples of domain flexibility in GT-Bs include Gram-positive bacterial enzymes *N*-acetyl-glucosamine transferase (MshA, retaining, EC: 2.4.1.250, CAZy family GT4), which mediates the first step of mycothiol biosynthesis [4], and TDP-epi-vancosaminyltransferase (GtfA, inverting, EC: 2.4.1.311, CAZy family GT1), involved in biosynthesis of the natural glycopeptide antibiotic chloroeremomycin [5]. Further evidence of conformational changes in GT-Bs upon substrate binding comes from intrinsic tryptophan fluorescence (ITF) experiments of the Gram-negative bacterial enzyme Heptosyltransferase I (HepI, inverting, EC: 2.4.99.B6, CAZy family GT9) [17,18]. HepI catalyzes the addition of the first heptose monosaccharide to the nascent polysaccharide core of the outer membrane lipopolysaccharide (LPS) [19,20,21,22]. Binding of the acceptor substrate analog (O-deacylated *E. coli* Kdo_2_-lipid A, ODLA) to HepI resulted in a spectral blue-shift of the enzyme’s fluorescence profile. Such a shift is indicative of associated structural changes that desolvate one or more tryptophan residues. The extent and degree of the HepI motion, however, cannot be explicitly described due to the nature of the ensemble averaged fluorescence experiments that were performed [17,18].

Some molecular dynamics simulations have been performed to date on GTs; however, these simulations have yet to elucidate the conformational transitions necessary for GT-B proteins to undergo the open-to-closed structural transition. The majority of the simulations to date were performed using protein structures built from homology models as their structural starting point due to the lack of available crystal structures for the desired proteins. Several GT-As, including GT MG517 from *Mycoplasma genitalium* [23], and LARGE1 from humans [24], together with two GT-Bs, GnT-V from humans [25] and HepIII from *Klebsiella pneumonia* [26], have been modeled and short molecular dynamics simulations have been performed to refine the structure and to corroborate or provide new insight for substrate/protein interactions. For those GTs that have crystal structures available, short (<300 ns) all atom or longer (10 µs) course grain molecular dynamic simulations have been performed. These simulations have largely focused upon substrate binding interactions (i.e., LgtC (GT-A), Gpgs (GT-A), and GumK (GT-B)) and in some cases, interactions of the protein with membranes (i.e., alMGS (GT-B) and PglH (GT-B) [2,27,28,29,30]. While multiple enzymes of both the GT-A and GT-B structural classes have been simulated, detailed analysis of the trajectories have only been performed in the GT-A structural scaffolds. Substrate binding and catalysis in GT-A, unlike GT-B, only involves rearrangement of loop regions, as the protein domains are continuous and largely unchanging over the course of the reaction.

We hypothesized that the evolutionary conserved ternary structure, chemical mechanism, and prerequisite domain flexibility required for function across inverting GT-Bs would result in conformational dynamics also being a conserved feature of these familial enzymes, despite low levels of sequence conservation (<30% sequence identity) between enzymes of different CAZy families. To test this, we characterize the type, degree, and order of the bound to unbound transition observed domain flexibility motions in HepI and GtfA as reported by microsecond molecular dynamics (MD) trajectories generated on the Anton1 supercomputer [31]. The advent of Anton1 has allowed for the atomistic examination of the dynamic behavior and associated properties of large biomolecular systems in explicit solvent at timescales that range from microseconds to sub-milliseconds [32,33,34,35]. The selection of the GT-B’s GtfA and HepI for MD study pertains to: (1) the availability of high resolution bound-form structures with no unresolved backbone or C_α_ atom coordinates; (2) a shared inversion catalytic mechanism; (3) pairwise sequence identity of 26.7%; and (4) a common catalytic core, discovered during this study, which has been evolutionary conserved in both sequence and three-dimensional (3D) space despite the divergent function of each enzyme. Our recent experimental and computational study has shown nearby positively charged residues within the enzyme active site play an essential role in its conformational transitions and the binding of its negatively charged substrates [36]. To further explore how distal residues may play a similar role, distal nonionizable residues were specifically identified from our simulations situated in the C-terminal domain with motions anticorrelated to positively charged active site residues in the *N*-terminal domain and mutagenesis was carried out to examine their effect on catalytic efficiency.

## 2. Results

### 2.1. Differential Dynamic Flexibilities in Open and Closed Configurations in Hepl and GtfA

The overall stability of tertiary structures of both GT-B systems was assessed by calculating the enzymes’ global Radius of Gyration (C_α_RGYR), C_α_ Root Mean Square Deviation (C_α_RMSD) and C_α_ Root Mean Square Fluctuation (C_α_RMSF). The computed C_α_RMSD for the binary complex structure of HepI shows an oscillatory behavior that is roughly 1 µs time scale, whereas short-time fluctuation is significant in a range between 1.5 and 3.5 Å (Figure S1). We attribute this long-time scale process to the overall domain–domain bending motions in the open configuration of the full protein. Of the structural ensembles represented by the full HepI trajectory, 11.5% of the frames report enzyme structures with C_α_RMSD values >3.0 Å, while 49.4% are between 2.0 and 3.0 Å. The C_α_RMSD for ternary complex GtfA, which adopts a closed configuration with respect to the two structural domains, shows an initial increase, which is leveled to an average value of about 2.7 Å after 750 ns. We note that while it takes a relatively longer time for the ternary complex GtfA to reach equilibrium, it is interesting to point out that the long-time oscillating motion has been quenched in the closed configuration. A locally weighted scatterplot smoothing (lowess) curve fit to the C_α_RMSD data shows the increase to be biphasic: an initial ~0.75 µs long phase of rapid, continuous rise in the C_α_RMSD followed by a final phase represented by steady fluctuations between 2.5 and 3.0 Å. Of the entire trajectory, 1.35% of the structures of GtfA report C_α_RMSD values >3.0 Å, while 78.15% have values spanning from 2.0 to 3.0 Å.

**Figure 2:**
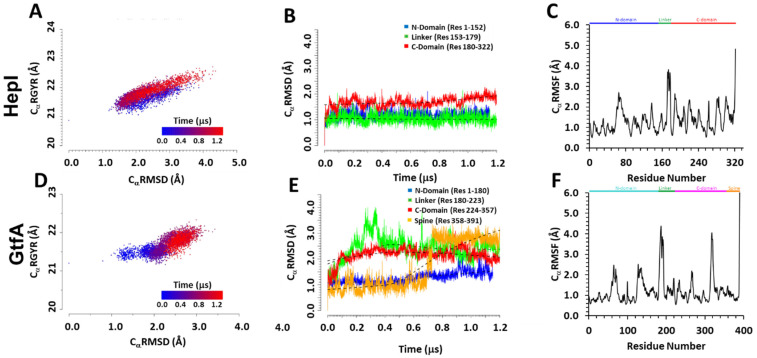
Computed radius of gyration (C_α_RMSD), root-mean-square deviation (C_α_RMSD), and root-mean-square fluctuation (C_α_RMSF) of alpha-carbon atoms in HepI (**A**–**C**) and GtfA (**D**–**F**). (**A**,**D**) The correlation between C_α_ RGYR and C_α_RMSD, highlighting the change in conformation population over the course of MD simulation from that determined at 0.0 (blue) to 1.2 μs (red); (**B**,**E**) the variations of C_α_RMSD divided into different glycosyltransferase domains over the course of the entire simulation. Black dotted curves represent lowess curves for each domain. (**C**,**F**) The C_α_RMSF plot with secondary structures, α-helix and β-sheet regions, highlighted in blue and yellow, respectively.

The C_α_RGYR data of HepI and GtfA (Figure 2A,D) demonstrate the change in compactness of each protein to highlight the opening of the closed structure GtfA structure as compared to the opened HepI. The plot continually exhibits different features for the binary open configuration and ternary closed form, respectively.

The correlation between computed radius of gyration and C_a_RMSD for HepI is simulation time-independent, with the same trend in spread and variation, which is consistent with the above assignment of an overall structural oscillation. The positive correlation of expanding radius of gyration with increased C_a_RMSD values is also consistent with the overall domain–domain breathing/bending motions. On the other hand, for the GtfA ternary complex, the radius of gyration is relatively independent with increasing C_a_RMSD in GtfA fluctuations before 0.5 μs, but as the system reached equilibrium in the later stages of simulation, a positive correlation between the two properties was observed as it should be. Importantly, the fluctuations of C_α_RGYR and C_α_RMSD in HepI cover broader ranges and reach greater values in both quantities than those in GtfA, indicating that the latter closed configuration is relatively more compact than that of the open configuration of HepI. The data suggest that changes in the compactness of the two enzymes are connected to the underlying global structural changes as measured by the C_α_RMSD. To further recapitulate the transition of the starting “closed” structure of GtfA to the “open” form, we evaluated the inter-domain distance between the *N-* and C-terminal domains and determined their probability density distribution over the course of the simulation (Supplemental Figure S6A,B). The observed bimodal distribution of GtfA showed a compact “closed” form with inter-domain distance of 9 Å and an enlarged “open” form with an inter-domain distance of 24 Å that coincides with the natural distribution of the starting “open” form of HepI.

### 2.2. Distinguishing Characters in Dynamic Fluctuations from Different Secondary Structures

To explore whether the data generated from the present simulations are representative of structural instability or physical microsecond-dynamic motions (e.g., loop restructuring, domain repositioning), the structural stability and dynamic rearrangements of the ***N***, ***L***inker, ***C***, and ***S***pine (GtfA only) domains were examined. To this end, we have determined (1) the C_α_RMSD of each domain individually (per-domain: *N*, *L*, *C*, and *S* C_α_RMSD) (Figure 2B,E), and (2) the C_α_RMSD of the specific secondary structural elements in each domain ([Domain]_α/β_-ssC_α_RMSD) (Figure S2). The data for these localized C_α_RMSD analyses were calculated with a common reference to the corresponding crystal structure coordinates that are aligned by the protein regions of interest. The resulting data report exclusively on the changes to the structure of the chosen protein segment(s). As such, the per-domain C_α_RMSD analysis functions as a macroscopic evaluation of the domain’s total structure, while the per secondary structure type C_α_RMSD serves as a microscopic assessment that focuses on changes to the underlying architecture of the domains.

The average HepI *C*_α_RMSD and ssRMSD for each domain fall under 2.0 Å and display a near-linear trend, with both the *N* and *C* domain β-sheet cores consistently having lower value, less fluctuating ssRMSD data than the enveloping α-helices. Unlike the HepI *N* and *L* domains, the HepI *C* domain is in a higher RMSD value range and steadily increases after ~0.6–0.7 µs (Figure 2B). The pattern observed in the C_α_RMSD beyond ~0.6 µs is both homologous to that of the C_β_-ssRMSD and matches the rise in the C_α_-ssRMSD during the same time period (Figure S2). The HepI ssRMSD of the β-sheet cores of *N* and *C* domains have lower averages and smaller fluctuations than the enveloping α-helices. The *N* and *L* domain RMSD are congruent and fit linearly to a lowess curve. On the other hand, with the exception of the *N* domain, increased variance is observed in the per-domain C_α_-RMSD GtfA. As in HepI, the C_α_-ssRMSD values all fall below 2.0 Å. The GtfA *N*-terminal domain C_α_-RMSD has a more constant, near-linear trend that begins to increase at ~0.6 µs. This sudden increase concurs with the start of the inward displacement of the *C*-terminal end of third *N*-terminal β-strand (*N*-β3; res: 50–52) towards the more central *N*-β2 (res: 30–35), as reported in the clearly defined jump at ~0.6 µs in the N_β_-ssRMSD. The start of a slight increase in the N_α_-ssRMSD was also observed at ~0.4 µs. This corresponds to the outward motion of residues 134–149, which comprise the *N*-terminal segment of *N*-α5 (res: 134–159).

The dynamic fluctuations of the spine domain of GtfA show that an initially fluctuating section in the computed *S*-C_α_RMSD initially is shifted to a tighter, slowly decreasing segment after approximately 0.7 µs (Figure 2E). Examination of the structures saved near the transition time clearly portrays the unwinding of the protein’s last three C-terminal residues (res: 389–391 on *S*-αII); no other significant alterations were observed in the rest of the spine’s backbone structure. GtfA *C* and *L* C_α_RMSD result in a near equivalent logarithmic dataset, peaking at ~0.2 µs and ~0.3 µs, respectively, followed by a period of steady decline. The C_α_-, C_β_-, or l_α_-ssC_α_RMSD data do not detail any significant structural changes to account for the shape of the *C* and *L* C_α_RMSD data (Figure 2B,E). With the exception of the global C_α_RMSD, none of the domain or ss-RMSD analyses consider loop (lp) or 3_10_-helix regions. From the C_α_ Root Mean Square Fluctuation (C_α_RMSF), the largest time-averaged atomic fluctuations (C_α_RMSF > 2.0 Å) in both enzymes primarily correspond to loop, 3_10_, and adjacent N/C-terminal secondary structure residues (Figure 2C,F). In HepI, these areas are: 63–70 (*N*-α3 C-term), 65–67 (*N*-lp5), 68–70 (*N*-α4 *N*-term) 136–137 (*N*-lp10), 188–191 (*C*-lp1 C-cap), 189–191 (*C*-3_10_1), 218–221 (*C*-lp2 C-cap), 219–221 (*C*-α2), 263 (*C*-α4 *N*-cap), 280–285 (*C*-lp7), 300–303 (*C*-3_10_3), and 321–322 (*C*-α5 C-term). In GtfA, the residues are: 64–65 and 70 (*N*-lp2), 127–128 (*N*-3_10_4), 183–193 (*L*-lp1), 266 and 268 (*C*-lp3), 315–322 (*C*-lp7), and 389–391 (*C*-α7 C-term). The locations of these high C_α_RMSF regions on the enzymes’ structures generally correspond to the side boundaries of the binding cavity on the *N* and *C* domains. The equivalence is solely spatial and does not always correlate to similar structural elements. A larger proportion of the high RMSF areas are positioned on the catalytic side of the cavity, primarily encompassing regions in the vicinity of the volume occupied (HepI) or predicted to be occupied (GtfA) by the sugar moiety of the cofactor. β-sheets in both systems consistently have the lowest C_α_RMSF values (<1.0 Å) and are data trough points. The α-helices demonstrate more fluctuation, with the high C_α_RMSF points corresponding to the helical N/C-terminal end residues.

### 2.3. Conformation Distribution Due to Low-Frequency Large Amplitude Motions

**Figure 3:**
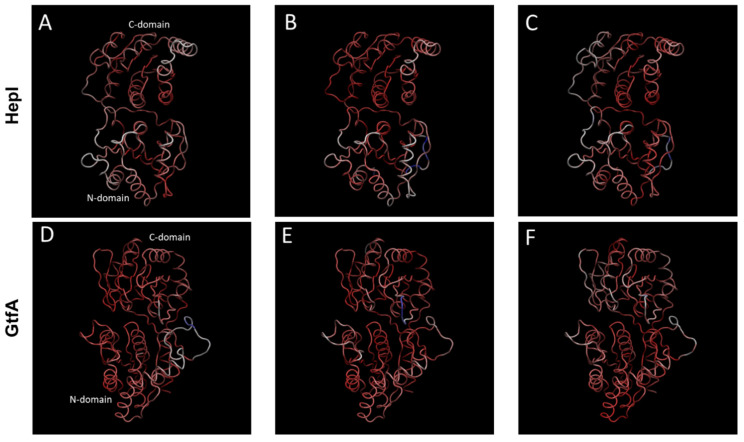
The first three principal components of quasiharmonic motions sampled by the 1.2 μs molecular dynamics trajectories for HepI (**A**–**C**) and for GtfA (**D**–**F**) (see Supplementary Figure S5 for animation). HepI and GtfA domains, C-domain, *N*-domain, and linker region are represented by white, blue, and green ribbons, respectively. These three large amplitude components account for 60.1 and 50.5% of dynamic motions, respectively, for HepI (top) and GtfA (bottom).

Principal component analyses (PCAs) of the trajectories reveal that large structural fluctuations of both systems are not limited to loop and 3_10_-helix movements (Figure 3).

We found that the values of 55.9 and 50.5%, respectively, of the overall structural variance from the HepI and GtfA simulations, are associated with the first three principal components (Figure S3). To better describe the underlying structural motions featured by each PC, an animated trajectory that interpolates between the extremes points of each principal component through an even number of time steps was created (Figure S4). The extreme points represent a single trajectory frame whose protein backbone arrangement (C_α_ coordinates) is projected onto a PC, reaching the farthest stretch displacement. The motions in the top three motions for both enzyme systems correspond to domain–domain movements hinged about the linker/spine region with slight variances between each principal component.

PC1 of HepI (PC1_H_, 35.3% structural variance), pertains to quasiharmonic motion, centered about the linker region, around residues 168–175 of the second loop of the linker region (*L*-lp2), with an antiparallel domain twist as the hinge moves. PC2_H_ (11.1% structural variance) corresponds to an antiparallel rotation of the *C* and *N* domains, hinged around linker residues 164–166 and 168–174 of *L*-lp2, identified from nonstructured regions of C_α_RMSF. PC3_H_ (9.5% structural variance), involves hinge motion with no twisting of domains, anchored about linker (164–165 [*L*-lp1], 166–169 [*L*-α1]) and *C* domain (180–184 [*C*-β1], 262 [*C*-β4], 263 and 266–267 [*C*-α6], 282–284 and 288–291 [*C*-lp7]) residues.

**Figure 4:**
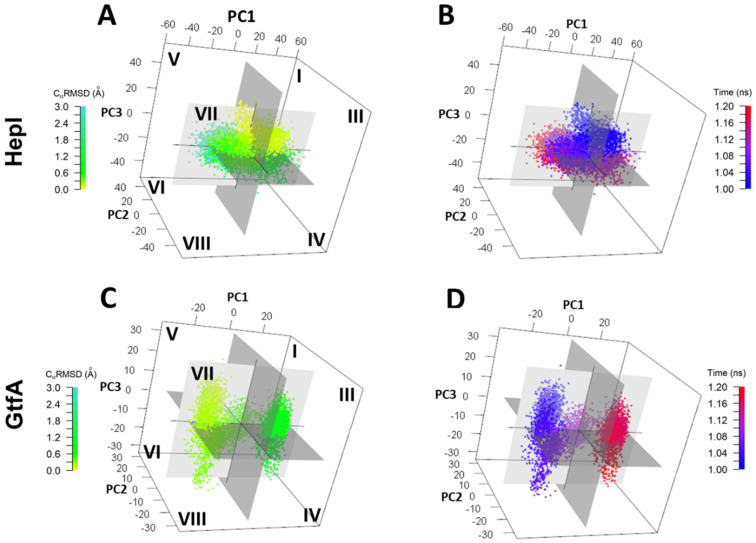
Conformation distribution of the top three principal components (3D-PCA) plotted against (**B**,**D**) C_a_RMSD (green to teal) and (**A**,**C**) time from 0.0 μs (blue) through 1.2 μs (red) for HepI (top) and Gtf A (bottom).

To observe the interplay of the dominant principal component motions of HepI over the trajectory we analyzed the top three principal components by time-clusters plotted in 3D space with relative PC coordinates defined as (±PC1, ±PC2, ±PC3) (Figure 4).

For HepI, although the global C_α_RMSD is periodic, the dominant principal components are not necessarily harmonic. The conformational dynamics of HepI system begins at octant III and ends in octant VIII. HepI crosses between negative and positive PC1 space six times chronologically over 1.2 microseconds, whereas it only crosses zero eigenvalue in PC2 and 3 once, indicating the motion of PC1 occurs in a faster timescale than the motions of PC2 and 3, respectively. In 3D space, HepI occupies each octant I-VIII for 8.3, 14.0, 11.4, 13.6, 11.4, 12.3, 13.2, and 16.1% of the trajectory, respectively (Table S1). There are, however, single points or segments of the trajectory that deviate from the above described progression and are found in an octant other than that of the time-cluster it would be affiliated with. Overall, these low-frequency large amplitude motions are consistent with the distribution of radius of gyration and C_α_RMSD shown in Figure 2A,E.

In PC1 of GtfA (PC1_G_, 33.5% structural variance), the motion is akin to the movement of the *N* and *C* domains observed in PC3_H_ (Figure S5C,D) with the loop moving perpendicularly from the N domain. The rotation is anchored on specific linker (177–179 [*N*-β6], 180–201 [*L*-lp1]) and spine residues (369–370 [*S*-3_10_1], 373–374 and 379–380 [*S*-α2]). PC2 of GtfA shows a rotational displacement of the *C* and N domains, next to the linker (181–194, 196–197, and 201–202 of *L*-lp1) and spine residues (372–376 and 378–379 [*S*-α2]) (Figure S5E). The movement in PC2_G_ is similar to the motion represented by PC2_H_. PC3_G_ displays the same domain motion as in PC2_H_. The rotational motion is hinged on linker (184–194 and 196–197 [*L*-lp1]) and spine (374–379 [*S*-α2]) residues (Figure S5F). GtfA begins in octant V and ends in octant III transitioning over positive and negative eigenvalues once for PC1, and two and three times for PC2 and PC3, respectively (Figure 4). GtfA occupies a larger proportion of the trajectory time in octants III and V (20.1 and 17.9%, respectively) and less time in octants I, IV, VI, and VII (7.1, 9.7, 6.1, 7.7%, respectively) (Table S1). By tracking the evolution of PC_G_ data clusters over time, the system’s progression through 3D octants of PC space are: 5 to 7 to 8 to 4 to 1 to 3. Another commonality between the two enzymes is the presence of structures in octants other than that expected, given its corresponding time value, but matches with C_α_RMSD and C_α_RGYR. The key observations are HepI dynamics are relatively time-independent, with even distributions in time, suggesting it is dynamically oscillating about the average. However, for GtfA, there is an equilibration time (first 0.6–0.7 microseconds), followed by equilibrium distribution, which is narrower in range than HepI. HepI has a larger scale motion of two domains while GtfA is in closed configuration and fluctuates relatively less frequently.

### 2.4. Dynamics Restriction Affecting Catalytic Efficiency from Site-Directed Mutations

**Figure 5:**
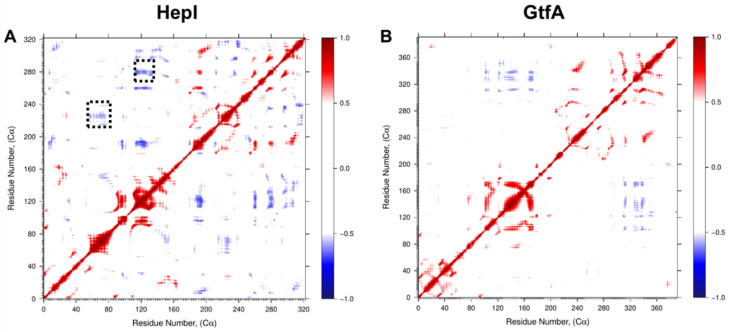
Dynamic C_α_ residue cross-correlation (DCC) of residue fluctuation over 1.2 µs MD simulation showing positively correlated (red), negatively correlated (blue), and uncorrelated (white) regions of (**A**) HepI and (**B**) GtfA. Dotted black boxes denote 60 and 120 s loop regions identified for mutagenesis.

Examination of the dynamic cross-correlation matrices (Figure 5) shows that residues near each other in their primary sequence demonstrate strong positively correlated motions, as illustrated by the deep red coloring on and near the diagonal of the matrix for both HepI and GtfA. Additionally, off-diagonal regions within the same domain predominantly demonstrate strong positively correlated motions, while off-diagonal regions that represent the relationship between the two domains (i.e., HepI *N*-terminal domain residues 1–152 with C-terminal domain residues 180–322) demonstrate predominantly negatively correlated motions.

**Figure 6:**
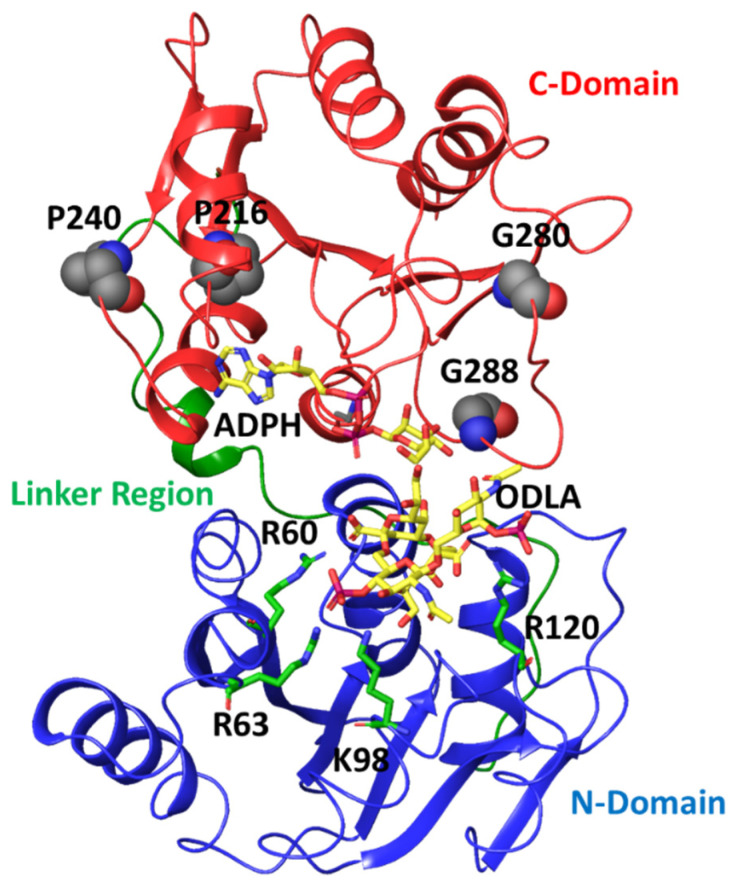
Crystal structure of HepI (PDB: 2H1H) with N domain, *C* domain and linker region colored in blue, red, green, respectively. The modeled ADPH and ODLA substrates (both in yellow) highlight the location of the catalytic site. The positively charged residues (R60, R63, K98 and R120) involved in FDLA binding are shown as stick representations. The four distal nonionizable residues (P216, P240, G280 and G288) located in the C-domain are shown in spacefill representation.

Focusing on correlations to residues in HepI that are known to participate in conformational changes associated with binding of the sugar acceptor ligand (including Lys 64, Arg 63 and Arg 120) [17,18,36], we can identify a series of glycine and proline residues distant from the binding sites of either substrate (>25 Å away from the binding site of the sugar acceptor but within 15 Å of the sugar donor binding site) with strong negative correlated motions (Pro 216, Pro 240, Gly 280 and Gly 288) (Figure 6). 

This lead us to hypothesize that these residues were necessary for sensing the binding of the sugar donor substrate (ADP-L-glycero-*D*-manno-heptose, ADPH), with Pro 216 and Pro 240 recognizing bind of the adenosine portion and Gly 280 and Gly 288 sensing the heptose portion. Due to the inherent flexibility of residues Gly 280 and Gly 288 (as well as the large degree of fluctuation observed for these residues over the course of the simulation; Figure 2C) and the loop in which they occupy lead to the hypothesis that these residues could play a role of differentiating between the bound sugar donor substrate and the nucleotide product, and critical for water exclusion from the active site. As part of our future directions, simulations of the liganded complex will be pursued to test this hypothesis. In the interim, experimental and computational studies were performed to investigate the role of these residues in catalysis. These glycine residues were individually mutated to prolines, and these proline residues were individually mutated to glycine, so as to yield mutant proteins with the greatest possible perturbation of their conformational flexibility at each position. The resultant proteins were then expressed, purified and kinetically characterized using high concentration of the sugar donor substrate (ADPH) while varying concentrations of the sugar acceptor substrate (ODLA which binds to the *N*-terminal residues that previously showed a strong degree of correlation to these Pro and Gly residues) to assess the impact of these residues on its binding (Table 1). 

**Figure 7:**
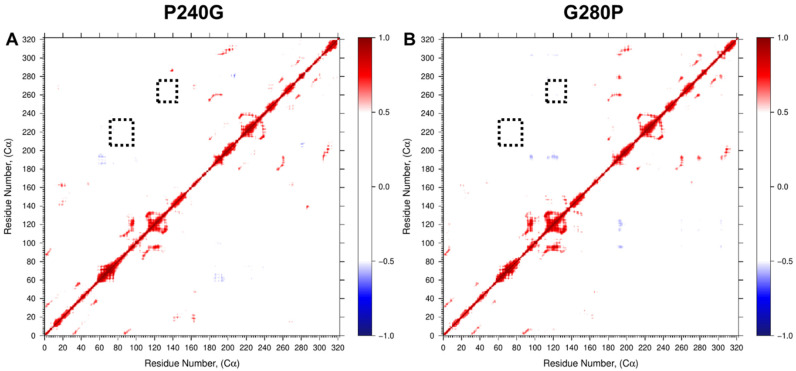
Dynamic cross-correlation (DCC) of residue fluctuation over 0.5 µs MD simulation of (**A**) P240G and (**B**) G280P HepI mutants showing loss of negatively correlated motion. Dotted black boxes denote 60 and 120 s loop regions identified for mutagenesis.

Each of the mutant HepI proteins exhibited modestly diminished *k*_cat_ values (with reductions of 3.1-fold or less as compared to wild-type HepI). Significantly, three of the mutants P216G, P240G and G280P all showed reduced *K*_m_ values (HepI•ODLA complex) to 1.6, 0.9 and 1.6 µM, respectively. The reduction in observed *K*_m_ in the mutant proteins leads to an overall enhanced catalytic efficiency (*k*_cat_/*K*_m_) relative to the wild-type enzyme HepI. To further establish how these mutants could have enhanced the observed catalytic efficiency, additional 0.5 ms MD simulations were carried out for P240G and G280P (the mutants with the largest experimental fold change of *K*_m_). Since these residues exhibited motions that were strong and anticorrelated to the residues identified previously by us as essential to ligand binding [36], mutation that removes this anticorrelated interaction as observed in our DCC analyses (Figure 7) would be expected to result in direct enhancement to ligand binding affinity.

## 3. Discussion

We demonstrate that microsecond simulations of these GTs led to trajectories that encompass a robust sampling of conformational substates in both enzymes, regardless of whether the starting protein structure was from a ligand-bound or unbound state. We perform MD statistical analyses and employ 3D principal component analysis (PCA) to highlight structural retention (global and by domain) during the course of the simulation, as well as identification of the conformational substates and their transitions along the bound to unbound pathway. We show these substates to correspond to domain flexibility (domain repositioning) events that depict the same hierarchy in both enzymes regardless of MD starting state (GtfA: closed bound-ternary; HepI: open bound-binary). Our observation of a hierarchy of substates is akin to similar processes reported via in vitro and/or in silico studies in myoglobin [37,38] and in adenylate kinase [39]. In those systems, the enzymes transition between substates with preferred directionality rather than by stochastic sampling. Whether the reported conformational directionality extends to familial enzymes of those systems remains to be fully determined.

For HepI, the predominance of per-domain RMSD values ≤ 2.0 Å highlight the structural permanence and stability of the domains. Structurally, the changes to the *C* domain starting at ~0.6 µs correspond to a slight outward displacement of the helical *N*-termini (*N*-cap and adjacent neighboring residues in both directions) of α-helices C-α2 (res: 219–231), C-α3 (res: 243–253), and C-α4 (res: 262–271). The *N*-termini of all three helices form part of the binding cavity. Specifically, the latter helix (C- α4) is positioned at the cavity midpoint, directly over the enzyme’s catalytic core on *N*-β1, while the *N*-termini of the two former helices are situated on the noncatalytic side of the binding cavity. In the crystal structure (PDB: 2H1H), these three regions associate with the adenine base of the sugar cofactor. Residue E222 of C-α2 forms hydrogen bonds with the 2′ and 3′ hydroxyls of the ribose. Residue M242, which is the amino acid right before the start of the *N*-terminal end of C-α3, forms a hydrogen bond between its backbone carbonyl oxygen and the amine of the adenosine base. In C-α4, the *N*-cap T262 hydrogen bonds to an α-phosphate oxygen of the diphosphate group. The same small outward motion is also evident from ~0.6 µs onward in C-β5 (res: 275–279) and C-β6 (res: 294–298). Both β-sheets are located on the catalytic side of the binding cavity.

Ultimately, despite obtaining 1.2 µs MD simulations of both enzymes, we were only able to observe the partial interconversion between the open and closed conformations that provided a glimpses into the principle motions along the path for catalysis (Figure 3 and Figure 4).Despite not realizing our full goal, the resulting trajectory data enabled us to (i) examine the root mean square fluctuation (RMSF) of residues, (ii) assess the dynamic cross-correlation (DCC) of the protein movements and (iii) identify the principal components of the motions of both HepI and GtfA. We noticed dramatic evolution of the C_α_RMSD for GtfA (closed with substrates removed) and modest changes in HepI (open unbound), consistent with our hypothesis that removal of the substrates will return the enzyme into its unbound state with an “open” active site for substrate binding. The C_α_RMSF analyses enabled the identification of dynamic loop regions of both proteins that were adjacent to the ligand binding sites that were suspected to be essential for ligand induced conformational changes. Subsequently, both the 60 and 120 s loops of the *N*-terminal domain (residues 1–152) of HepI were shown to be critical for substrate binding experimentally, which has also been subsequently confirmed by crystallography [2,3,40].

The DCC analyses (Figure 5) helped reveal that both proteins have a high degree of negatively correlated motions between the two domains, consistent with the enzyme practicing the open-to-closed motions even in the absence of sufficient time to perform the complete interconversion of those two states. To inquire whether dynamical motion in the unbound state plays any important role in catalysis, examination of the DCC matrices, specifically at the 60 and 120 s loops identified above, was carried out to reveal that there were numerous proline and glycine residues within the HepI C-terminal domain (residues 180–322; Table S1) that exhibited negatively correlated movements with these ligand binding residues. Since glycine and proline are known to exhibit dynamics that are distinct from other amino acids due to the lack of substitution at C-α (as with glycine) or the cyclical nature of the substitution at C-α (as with proline), and as others have noted their involvement in protein flexibility and protein folding [41,42], we mutated these residues to test the importance of their conformational variability on the overall behavior of the protein. We hypothesized that the rearrangement of the 60 and 120 s loops might communicate substrate occupancy of the *N*-terminal domain to the C-terminus (and visa-versa), and recent mutagenesis of these residues (where the glycines and prolines were interchanged; Table 1). Examination of the DCC for the P240G and G280P mutants reveals that the inter-domain negatively correlated motions observed in the WT for these residues are absent in the mutants (Figure 5 and Figure 7), while the resulting proteins bind ~10 times more tightly to the sugar acceptor in the *N*-terminal domain. The most pronounced effect was observed in the P240G mutant, which had nanomolar binding affinity for the sugar acceptor substrate, ODLA, and an overall catalytic efficiency of 2.0 × 10^5^. P240G and the other mutated residues located in the opposing C-terminal domain are all >25 Å away from the ligand binding site for the sugar acceptor in the *N*-domain, yet their mutation to alter their conformational lability enhances the enzyme’s ability to adopt the Michaelis complex with ODLA by an unknown mechanism. Our initial hypothesis was that these mutants yield a reduction in fluctuation in the 120, 240 and 280 s regions which may allow for a preordering of the enzyme into a conformation akin to that needed for the Michaelis complexes; however, this hypothesis requires further simulations of liganded complexes which is beyond the scope of this work. Interestingly, this finding in HepI supports the conclusions by Warshel and coworkers [43,44] that protein dynamics are primarily responsible for assisting the enzyme in adopting a state where chemistry can occur, and not in yielding a dynamical rate enhancement. Lastly, the analysis of principle components 1–3 of both HepI and GtfA (Figure 3 and Figure 4) revealed that despite starting in different conformations (HepI open and GtfA closed), the two proteins maintain the similar primary principle components and have a shared conformational hierarchy. While further simulations at longer times and with liganded complexes are needed to fully understand the role of dynamics in catalyzing these glycosyltransfer reactions, the results to date suggest to us that the overall dynamics of these proteins are a conserved feature of the GT-B structural scaffold, even when the proteins are evolutionarily diverged with little sequence similarity.

## 4. Material and Methods

### 4.1. Multiple Sequence Alignment (MSA)

The MSA was generated by filtering the HepI (UniProtKB: P24173) and GtfA (UniProtKB: P96558) sequences on the European Bioinformatics Institutes (EMBLEBI) Clustal Omega MSA web server with default settings. The resulting MSA was visualized and sequence identity conservation was determined using the Unipro UGENE (v. 1.14.2) bioinformatics software.

### 4.2. Molecular Dynamics (MD) Simulations

Protein systems are built and equilibrated from their respective crystal structure coordinates (PDB Databank) using the CHARMM MD simulation program (v. c38a2) and the CHARMM c36 all-atom force field with CMAP corrections. All selected crystal structures met the following criteria: resolution ≤ 2.5 Å, R-Factor ≤ 0.2, and R-Free ≤ 0.30. Missing atoms and hydrogens were built using CHARMM. Protonation states of charged residues were determined by means of pK_a_ calculations (H++ server) and by examination of the surrounding hydrogen-bonding environment. For neutral histidine residues, proton positions (δ or ε) were assigned according to hydrogen-bond donating and accepting patterns. Unbound systems were set up by ligand exclusion from the respective open-binary (HepI, PDB: 2H1H: Chain A) and closed-ternary (GtfA, PDB:1PN3: Chain A) structures. Both systems were solvated in a cubic box of water molecules described by the TIP3P model; the MMTSB toolset was used. Crystal water molecules were retained, and depending on the system, sodium or chloride counter-ions were added to ensure electrostatic neutrality. The SHAKE algorithm [45] was applied to all bonds involving hydrogen atoms, which eliminates the vibrational frequencies of fast moving bonds consisting of hydrogen atoms to reduce the communication frequency of the computed forces in the parallelization of MD, thus, enabling the use of longer time steps to integrate the equations of motions [46,47]. Close contacts were removed by energy minimization using 25–50 steps of Steepest Descent minimization followed by 25–50 steps of adopted basis Newton-Raphson Method minimization, in which the coordinates of heavy atoms of the protein backbone, the ligand, and crystal water were held fixed. Careful consideration was placed on retention of the overall protein structure via RMSD of C_α_ atoms. Harmonic restraints on all protein heavy atoms (100 kcal/mol) and fixed constraints on ligand heavy atoms was maintained throughout initial equilibration, which included: heating to 300 K with gradual scaling of temperature by 0.2 K every 100 femtoseconds (fs); application of the NPT ensemble (isobaric-isothermal) with Nosé-Hoover temperature control at 300 K for several ns until the reported pressure was consistently near 1 atm. Dynamics were propagated with the Leapfrog integrator with a time-step of 2 fs. Van der Waals forces were truncated with a switching function between 11 and 12 Å. Particle Mesh Ewald was used to model long-range electrostatic interactions with a real-space cutoff of 12 Å and a κ value of 0.333 Å; the grid space was set to be about 1 Å. Restraints on protein heavy atoms were then gradually released in a radial fashion (side-chains first, then backbone) from a defined point in the catalytic center using the NAMD simulation package (v. 2.8) for 10 ns. Final velocities and equilibrated coordinates were used to generate the necessary Anton1 input files (ark files) using the DESRES provided guesser scripts. Dynamics simulations were propagated using the RESPA integrator with a time-step of 2 fs. The NPT ensemble at 1 atm and 300 K was used with the Berendsen thermostat/barostat. Production was performed using 512 nodes of the Anton1 machine with structures saved in every 240 picoseconds during our allocation period. All simulation analyses were carried out using Bio3D [48] package in R. C_α_ root-mean-square deviation (C_α_RMSD), C_α_ root-mean-square fluctuation (C_α_RMSF), C_α_ radius of gyration (C_α_RGYR) and inter-domain distances were evaluated to determine the conformational changes over the course of microsecond simulation. Dynamic cross-correlation (DCC) and principal component analysis (PCA) were performed to determine the intramolecular “cross-talks” between domains and principal motions observed throughout the simulation. The follow up 0.5 μs simulations of HepI P240G and G280P mutants, after our Anton1 allocation period ended, were performed with Gromacs simulation package with the same forcefield and MD simulation conditions on Linux workstations with NVIDIA GPU.

### 4.3. Site-Directed Mutagenesis of HepI from E. coli K12

All materials, solvents, competent cells were obtained as previously reported [17,18,36]. The Q5 High-Fidelity PCR kit from New England Biolabs was used according to its instructions to perform mutagenesis using *E. coli K*-*12* strain MB1760 960 bp HepI gene subcloned into pTOM-15b. New England Biolabs thermocycling conditions for routine PCR was used and the amplified DNA was then transformed into XL10-Gold competent cells, which were then incubated in a 5 mL LB/AMP overnight growth for plasmid DNA extraction by miniprep. Once the mutation was confirmed by sequence alignment with HepI wild-type DNA and the respective primers for each mutant, the purified plasmid was transformed into BL21-AI cells as per Agilent’s instructions.

P216G Forward Primer: gcgcgccccacccaagtttaatccgtattcctg
P216G Reverse Primer: caggaatacggattaaacttgggtggggcgcgc
P240G Forward Primer: gttgaagtattgggcaagatgagtctggaaggcgttg
P240G Reverse Primer: caacgccttccagactcatcttgcccaatacttcaac
G280P Forward Primer: ggatagacccaatatcacggtttatccgccaaccgatccg
G280P Reverse Primer: cggatcggttggcggataaaccgtgatattgggtctatcc
G288P Forward Primer: ccgggattaattcctgggtatgggaagaatcagatggtatgtagggctcc
G288P Reverse Primer: ggagccctacataccatctgattcttcccatacccaggaattaatcccgg

### 4.4. Mutant Protein Expression and Purification

All buffers for SDS-PAGE gels and protein purification protocols are the same as for the wild-type HepI, as reported in the previous literature [29]. All mutants transformed into BL21-AI including WT were grown and expressed with the same conditions as described previously [17,18,36]. Briefly, cells were harvested, resuspended in 20 mL bind buffer per liter of growth (20 mM HEPES, 1 µM imidazole, 500 mM NaCl, pH = 7.5) on ice and lysozyme was added. The cells were then homogenized for 4–8 cycles and the lysate was clarified by centrifugation at 13,000 rpm for 1 h. The lysate was purified via FPLC with parameters described previously. Monitoring of 280 nm was used to identify fractions containing HepI and those fractions were then pooled and concentrated. The concentrated protein was desalted into a storage buffer of 100 mM HEPES, 1 M KCl, pH 7.5, using a BioRad P6 polyacrylamide SEC column. The fractions containing protein were then concentrated and precipitated in an equal volume of saturated ammonium sulfate and stored in amber vials at 4 °C.

### 4.5. HepI Mutant Kinetics Characterization

HepI substrates were prepared as previously described [36] for kinetics analysis of HepI and its mutants. Steady-state kinetic data were collected using a UV–Vis spectrophotometric technique of a pyruvate kinase-lactate dehydrogenase coupled assay which monitors the reduction in NADH overtime upon release of ADP. Data collected were analyzed using the Michaelis–Menten approximation and reported in Table 1.

## 5. Conclusions

This is the first ever all atom, microsecond MD simulation of GT-B glycosytransferases, and the study suggests that C_α_RMSD by itself is not a good test to determine structural stability at higher timeframes, which aligns with other work utilizing MD to simulate multidomain proteins connected by linker regions [49]. At these extended timescales, the simulations need to be examined for each individual domain and require domain-specific C_α_RMSD analysis. The simulations of these two distantly related, but structurally conserved glycosyltransferases have given us insight into how the structural scaffold itself, and not the primary amino acid sequences, control the protein dynamic modes. The interconversion processes are a collection of smaller, localized motions, rather than a global, whole domain motion around a hinge region, highlighting the complexities of the protein dynamics. HepI and GtfA are evolutionarily diverged in their primary sequence yet maintain a conservation of protein dynamic modes required for substrate binding, an aspartate residue involved in catalysis and residues for stabilization of the conserved oxocarbenium ion intermediate. The conformational directionality and the chemical environment of the active site is a preserved feature of these structures and is encoded in the fold rather than the sequence, perhaps illustrating why evolution of this structural fold has been recapitulated to produce a series of enzymes catalyzing sugar transfer reactions. Additionally, the observation that mutagenesis of these distal nonionizable residues which exhibit anticorrelated motions to those residues that are important for substrate binding suggests that the enzyme dynamics for HepI are enabling the preordering of the enzyme into the Michaelis complex and not chemistry. While dynamic motions are often considered to be critical for traversing activation energy barriers, in this case this mutagenesis data are consistent with the theory that dynamic modes enable the electrostatic organization of the Michaelis complex and do not make a significant contribution to catalysis.

## Figures and Tables

**Table 1:** Kinetic constants for the site-directed mutagenesis HepI.

HepI	k_cat_ (s^−1^)	Fold Change	K_M_ (M)	Fold Change	k_cat/_k_M_
Wild type	0.59 ± 0.09	-	8.5 ± 3.6	-	6.8 × 10^4^
P216G	0.21 ± 0.02	2.8 ↓	1.6 ± 0.6	5.6 ↓	1.3 × 10^5^
P240G	0.19 ± 0.01	3.1 ↓	0.9 ± 0.3	10 ↓	2.0 × 10^5^
G280P	0.21 ± 0.02	2.8 ↓	1.5 ± 0.5	6.0 ↓	1.3 × 10^5^
G288P	0.48 ± 0.07	1.2 ↓	10.2 ± 0.4	1.1 ↑	4.7 × 10^4^

## Data Availability

Not applicable.

## Supplementary Materials

The supplementary materials are available online at https://www.mdpi.com/article/10.3390/ijms22094619/s1
