# Supplementary material for: Conserved Conformational Hierarchy across Functionally Divergent Glycosyltransferases of the GT-B Structural Superfamily as Determined from Microsecond Molecular Dynamics
Source: Int J Mol Sci. 2021 Apr 28;22(9):4619. doi: 10.3390/ijms22094619 (PMC8124905; doi:10.3390/ijms22094619)
Supplement: Supplementary file 1 [file ijms-22-04619-s001.zip › ijms-1193544-supplementary.pptx]

## Slide 1
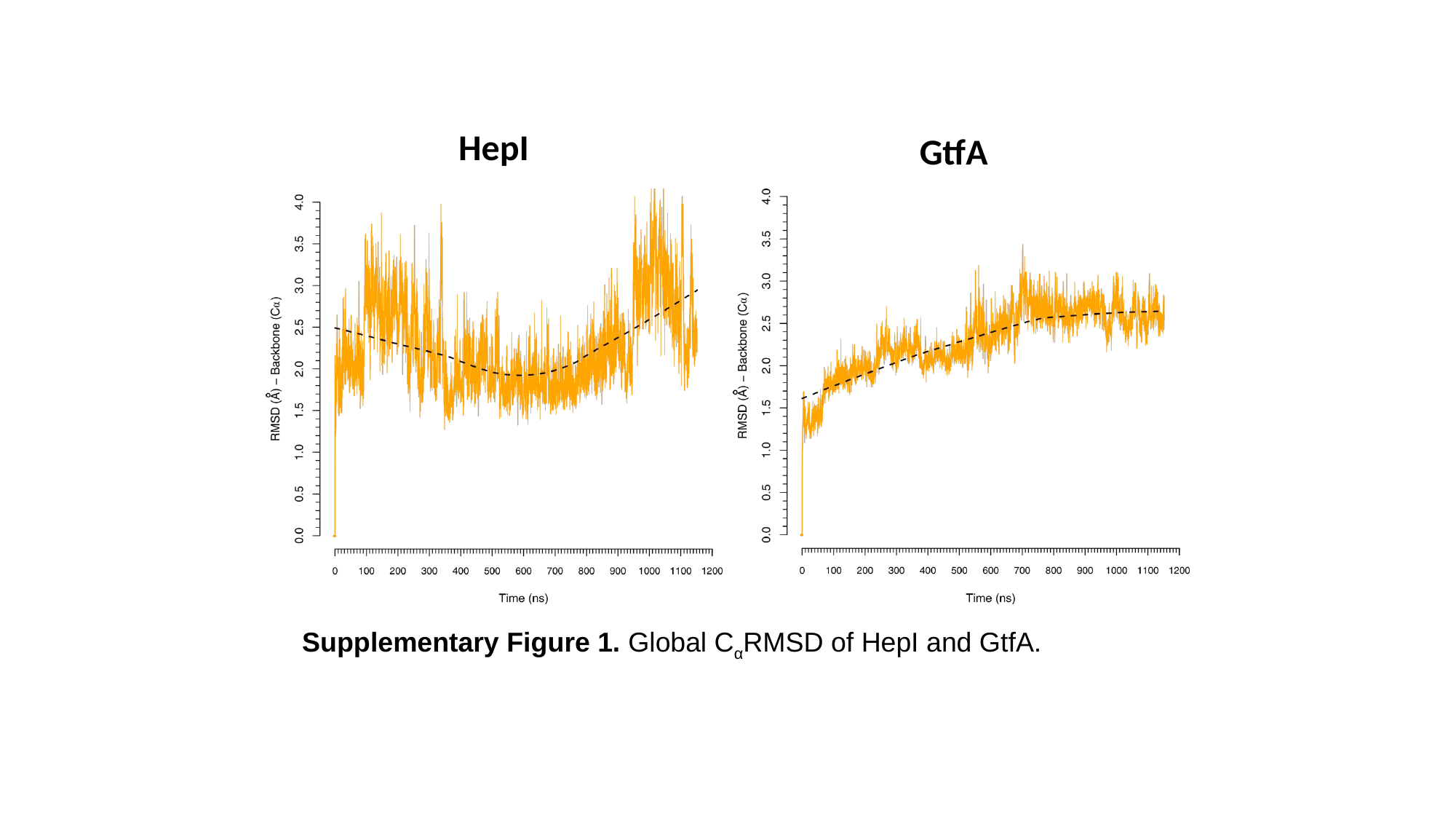

HepI
GtfA
Supplementary Figure 1. Global CαRMSD of HepI and GtfA.

## Slide 2
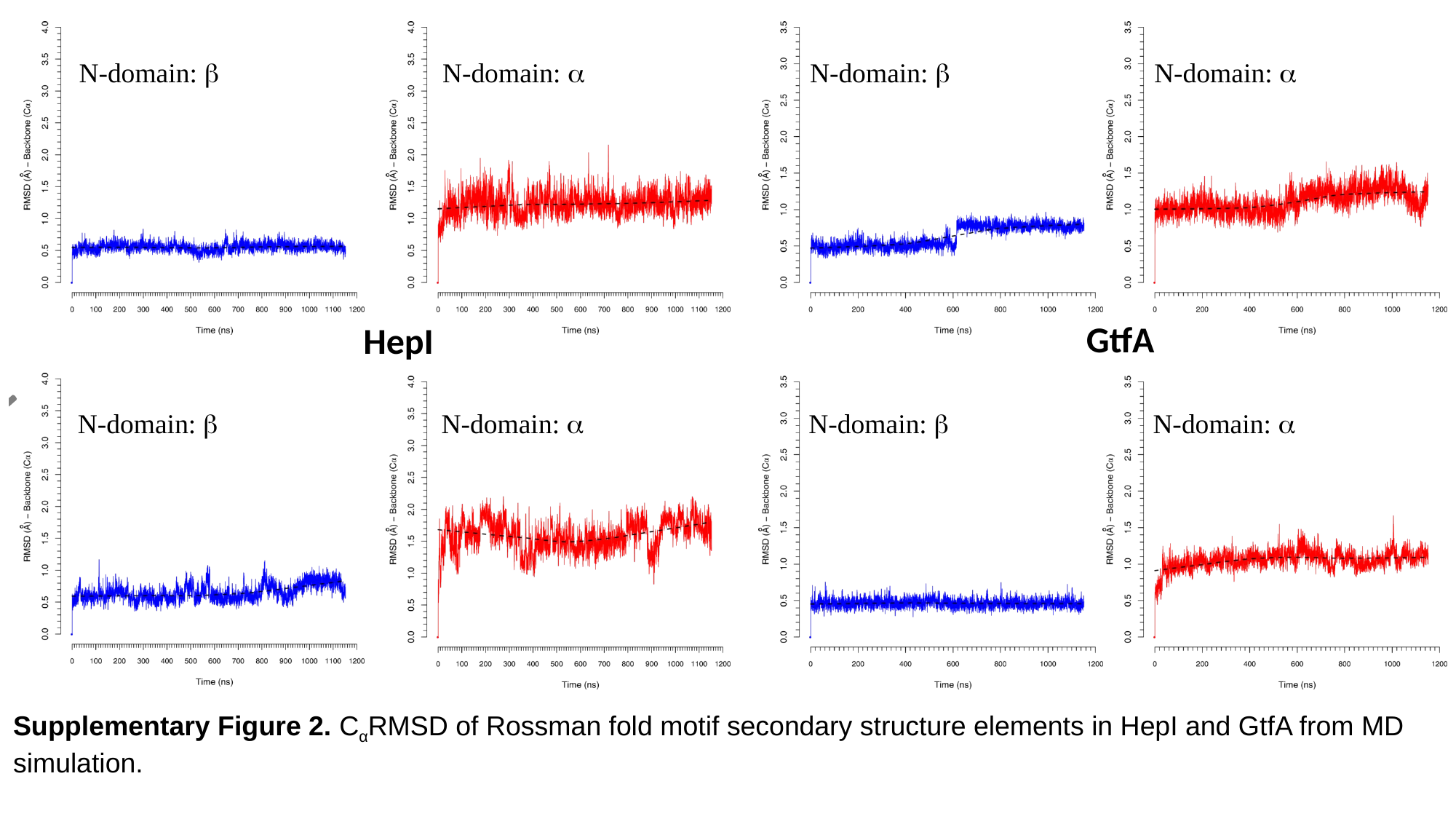

N-domain: b
N-domain: a
HepI
N-domain: b
N-domain: a
N-domain: b
N-domain: a
GtfA
N-domain: b
N-domain: a
Supplementary Figure 2. CαRMSD of Rossman fold motif secondary structure elements in HepI and GtfA from MD simulation.

## Slide 3
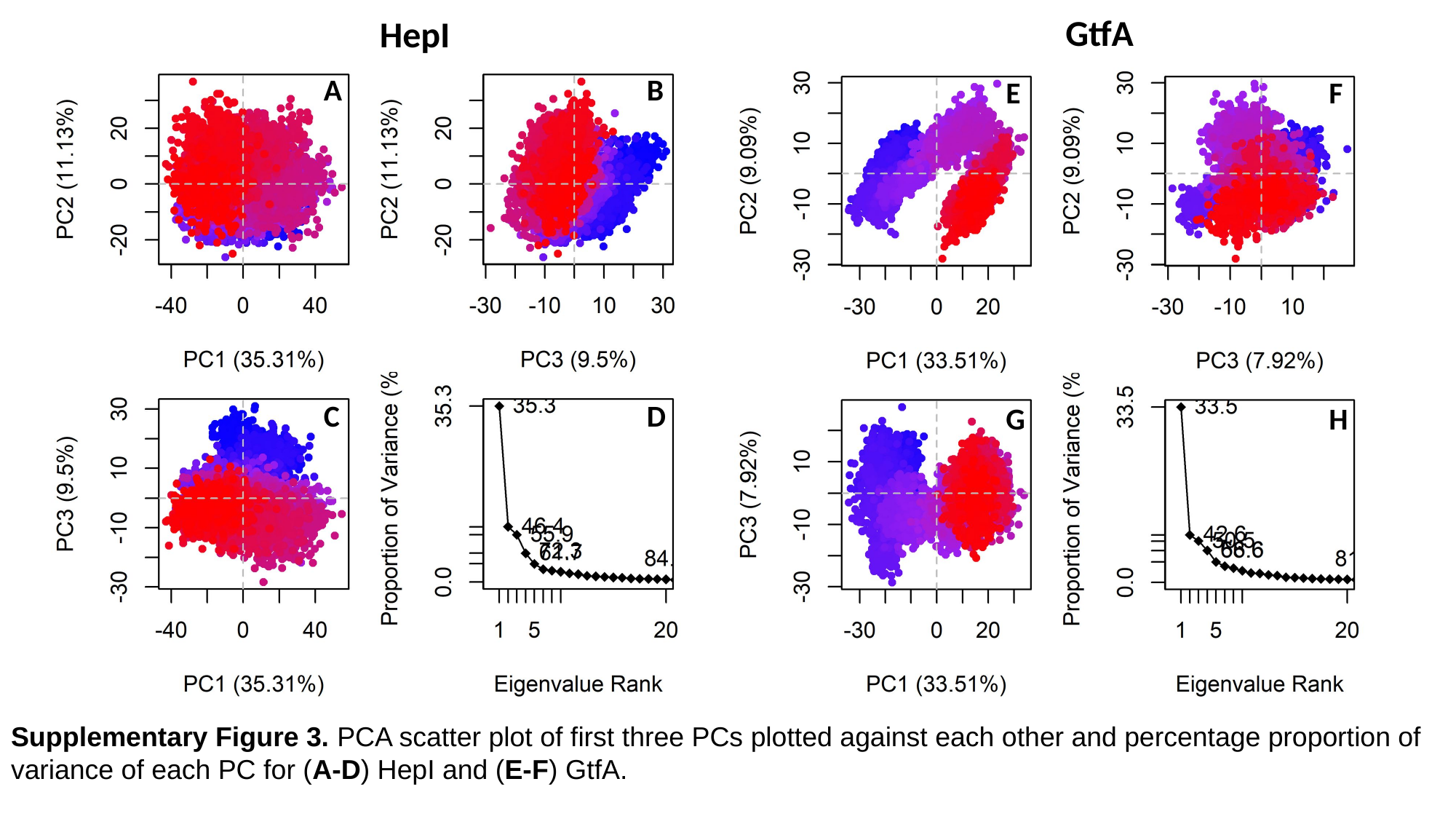

GtfA
E
F
G
H
HepI
A
B
C
D
Supplementary Figure 3. PCA scatter plot of first three PCs plotted against each other and percentage proportion of variance of each PC for (A-D) HepI and (E-F) GtfA.

## Slide 4
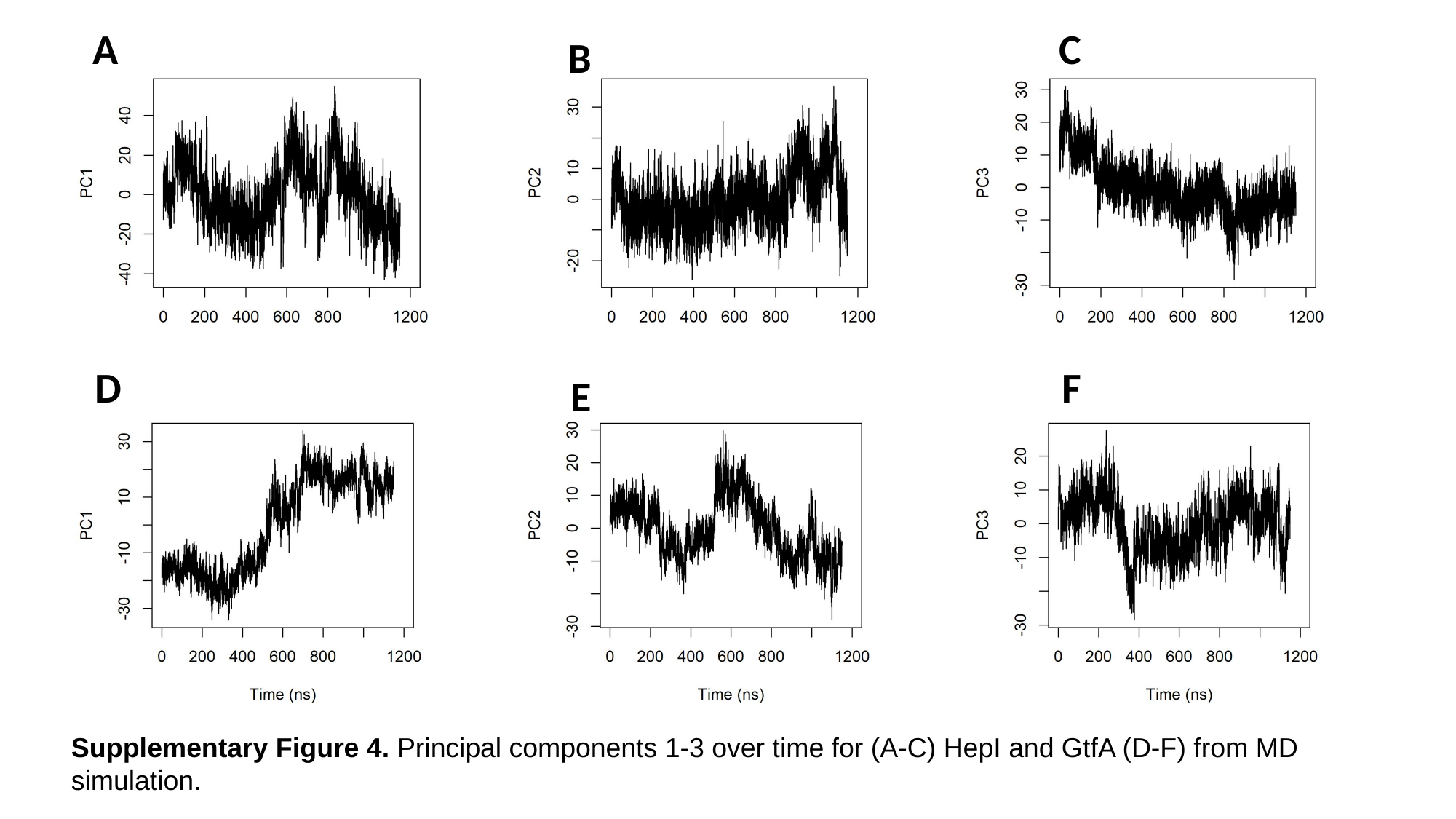

A
C
B
D
F
E
Supplementary Figure 4. Principal components 1-3 over time for (A-C) HepI and GtfA (D-F) from MD simulation.

## Slide 5
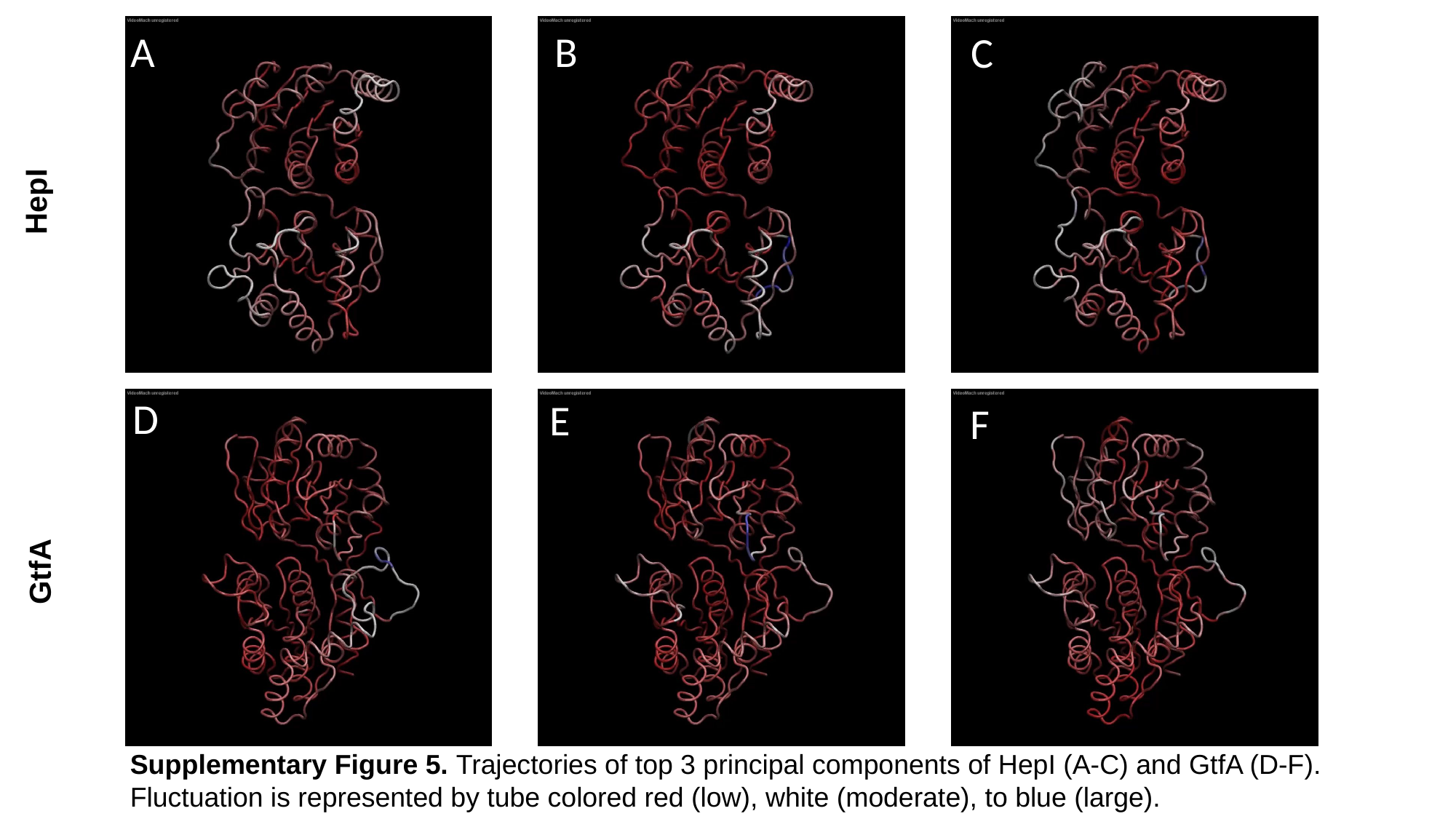

B
A
C
HepI
D
E
F
GtfA
Supplementary Figure 5. Trajectories of top 3 principal components of HepI (A-C) and GtfA (D-F). Fluctuation is represented by tube colored red (low), white (moderate), to blue (large).

## Slide 6
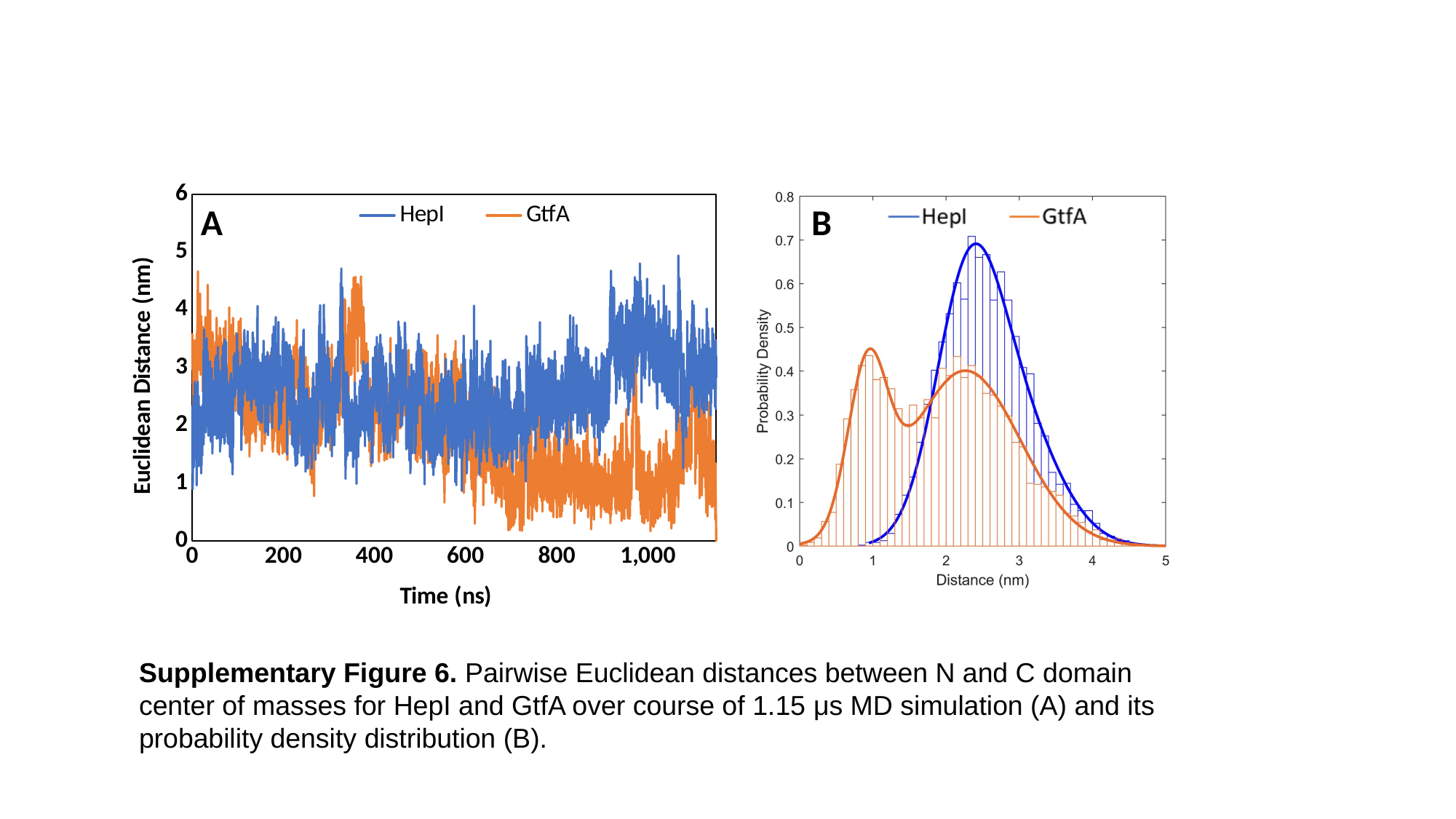

### Chart
| Category | | |
|---|---|---|A
B
Supplementary Figure 6. Pairwise Euclidean distances between N and C domain center of masses for HepI and GtfA over course of 1.15 μs MD simulation (A) and its
probability density distribution (B).

## Slide 7
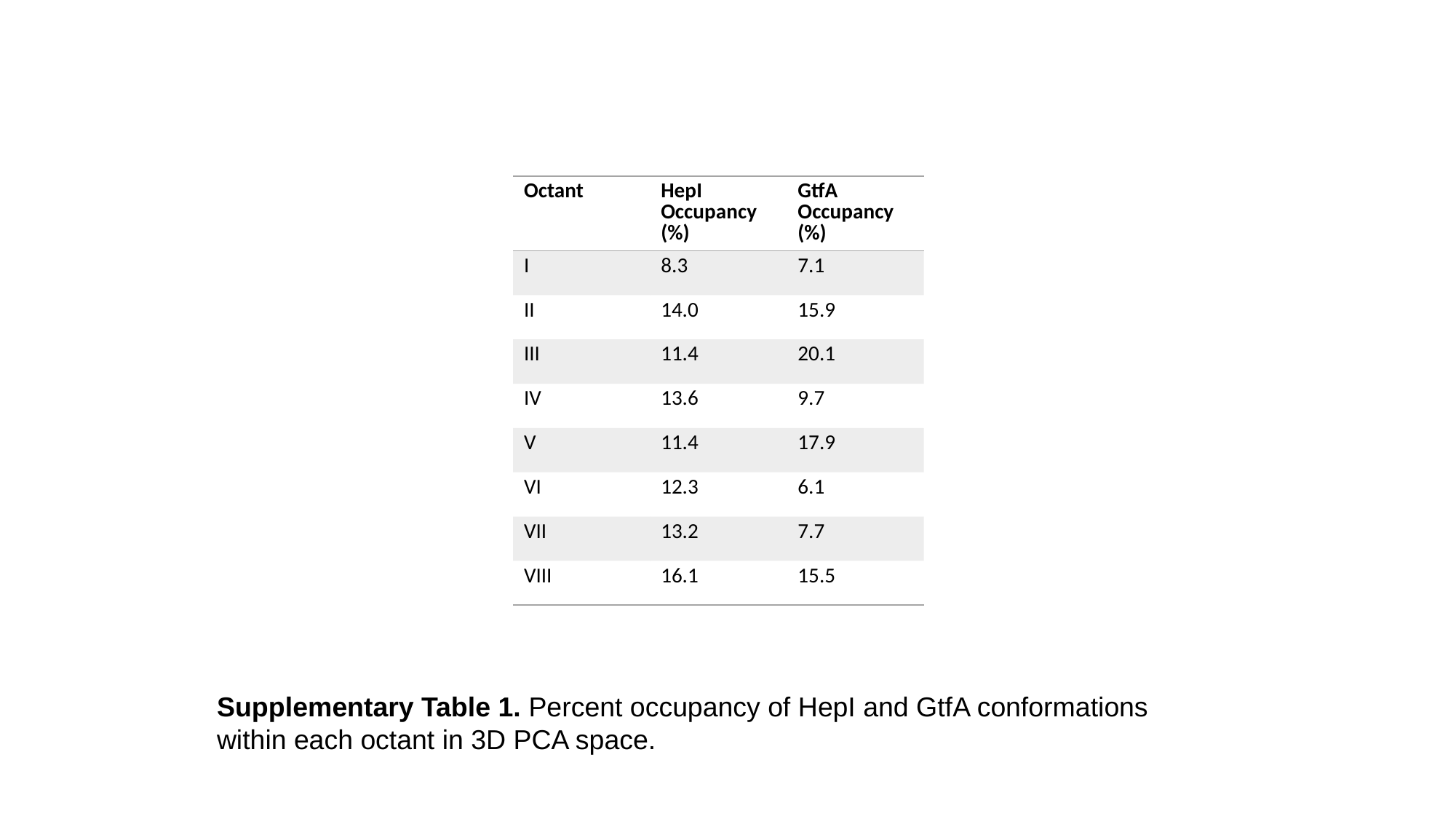

| Octant | HepI Occupancy (%) | GtfA Occupancy (%) |
| --- | --- | --- |
| I | 8.3 | 7.1 |
| II | 14.0 | 15.9 |
| III | 11.4 | 20.1 |
| IV | 13.6 | 9.7 |
| V | 11.4 | 17.9 |
| VI | 12.3 | 6.1 |
| VII | 13.2 | 7.7 |
| VIII | 16.1 | 15.5 |
Supplementary Table 1. Percent occupancy of HepI and GtfA conformations within each octant in 3D PCA space.
